# Distribution and Spread of the Mobilized RND Efflux Pump Gene Cluster *tmexCD-toprJ* in Klebsiella pneumoniae from Different Sources

**DOI:** 10.1128/spectrum.05364-22

**Published:** 2023-06-28

**Authors:** Lin Sun, Hanyun Wang, Nan Meng, Zhenyu Wang, Guiling Li, Xinan Jiao, Jing Wang

**Affiliations:** a Jiangsu Key Laboratory of Zoonosis, Jiangsu Co-Innovation Center for Prevention and Control of Important Animal Infectious Diseases and Zoonoses, Yangzhou University, Yangzhou, China; b Key Laboratory of Prevention and Control of Biological Hazard Factors (Animal Origin) for Agrifood Safety and Quality, Ministry of Agriculture of China, Yangzhou University, Yangzhou, China; c Department of Laboratory Medicine, Clinical Medical College, Yangzhou University, Yangzhou, China; d Clinical Testing Center, Northern Jiangsu People’s Hospital, Yangzhou, China; University of Nebraska—Lincoln

**Keywords:** tigecycline resistance, *Klebsiella pneumoniae*, efflux pump, *tmexCD-toprJ*

## Abstract

In this study, we screened the *tmexCD-toprJ* gene cluster among 1,541 samples obtained from patients, healthy individuals, companion animals, pigs, chicken, and pork and chicken meat in Yangzhou, China. As a result, nine strains from humans, animals, and foods were positive for *tmexCD1-toprJ1*, which was located on plasmids or the chromosome. Seven different sequence types (STs) were identified, including ST15 (*n* = 2), ST580, ST1944, ST2294, ST5982, ST6262 (*n* = 2), and ST6265. All the positive strains were clustered into two distinct clades, and they shared a 24,087-bp core structure of *tmexCD1-toprJ1*, bounded by IS*26* in the same orientation. IS*26* could facilitate rapid and wide dissemination of *tmexCD1-toprJ1* in *Enterobacteriaceae* from various sources.

**IMPORTANCE** Tigecycline has been regarded as one of the last-resort antibiotics available for the treatment of infections caused by carbapenem-resistant *Enterobacterales*. The plasmid-mediated resistance-nodulation-division–type efflux pump gene cluster *tmexCD*-*toprJ* is a newly identified tigecycline resistance determinant. In this study, we revealed that *tmexCD-toprJ* has disseminated among Klebsiella pneumoniae strains from poultry, food markets, and patients. It is critical to strengthen continuous monitoring, and control measures should be implemented to prevent the further dissemination of *tmexCD-toprJ*.

## OBSERVATION

Carbapenem-resistant *Enterobacterales* (CRE) is an urgent and growing threat to the global public health (1). Tigecycline has been regarded as one of the last-resort antibiotics available for the treatment of infections caused by CRE. However, tigecycline resistance has also emerged. The plasmid-mediated resistance-nodulation-division (RND)–type efflux pump gene cluster *tmexCD*-*toprJ* is a newly identified tigecycline resistance determinant (2). *tmexCD1*-*toprJ1* was first identified in Klebsiella pneumoniae isolated from chicken feces (2), and subsequently *tmexCD2-toprJ2* was identified from a clinical Raoultella ornithinolytica isolate (3), *tmexCD3-toprJ3* in Proteus mirabilis was isolated from swine feces (4), and *tmexCD4-toprJ4* was found in Klebsiella quasipneumoniae and Enterobacter roggenkampii isolated from chicken meat and environmental samples, respectively (5). A new nationwide surveillance in China revealed that *tmexCD-toprJ* has disseminated among diverse species of clinical pathogens, including Pseudomonas spp., Klebsiella spp., *Aeromonas* spp., and Proteus spp. (6). In this study, we report our investigation of *tmexCD-toprJ* in Klebsiella pneumoniae strains isolated from different sources in Yangzhou, China.

A total of 35 pig fecal samples were collected from 3 farms; 206 pet fecal samples were collected from 3 pet hospitals; 235 meat samples, including 117 pork and 118 chicken meat, were obtained from 2 local farmer markets; and 158 chicken intestinal content samples were collected from 4 slaughterhouses. In addition, 309 human feces samples were acquired from healthy donors. All the samples were gathered from June 2021 to September 2022 in Yangzhou, Jiangsu Province, China (see Table S1 in the supplemental material). Simultaneously, 598 nonrepetitive clinical K. pneumoniae isolates were collected from 2 tertiary hospitals in Yangzhou. All samples were enriched in Luria-Bertani medium for 18 h and then inoculated onto MacConkey inositol adonitol carbenicillin agar (Haibo, Qingdao, China) containing tigecycline (2 mg/liter). Species identification was performed using matrix-assisted laser desorption ionization–time-of-flight mass spectrometry (Bruker Daltonik GmbH, Bremen, Germany). The isolates were further screened for the *tmexCD-toprJ* gene cluster by PCR and Sanger sequencing as previously described (2). Nine strains from humans (*n* = 1), animals (*n* = 6), and foods (*n* = 2) were positive for *tmexCD1-toprJ1* (Table S1). Antibiotic susceptibility testing was performed using the agar dilution method or broth microdilution method (limited to colistin and tigecycline). The results were interpreted according to the 2020 Clinical and Laboratory Standards Institute guidelines (document M100-S30), except the breakpoints of streptomycin, tigecycline, and florfenicol were interpreted according to European Committee on Antimicrobial Susceptibility Testing guidelines (https://www.eucast.org/). Nine *tmexCD1-toprJ1*-positive strains exhibited resistance to tigecycline (MIC, ≥4 mg/liter) and were also resistant to ampicillin, cefazolin, gentamicin, amikacin, streptomycin, tetracycline, chloramphenicol, florfenicol, nalidixic acid, ciprofloxacin, and sulfamethoxazole-trimethoprim. Resistance to colistin (MIC, ≥4 mg/liter) was observed in YZ22PK089 (Table 1). Only one *tmexCD-toprJ*-positive strain, YZ22CK024, could transfer tigecycline resistance to Escherichia coli C600 by conjugation (Table S2).

**TABLE 1:** Characterization of *tmexCD1-toprJ1*-positive Klebsiella pneumoniae isolates^*a*^

Strain	Source	MIC (mg/liter)^*a*^																	Resistance genes	Location of *tmexCD1-toprJ1*
		ST	AMP	CFZ	CTX	MEM	GEN	AMK	STR	TET	TIL	CHL	FFC	NAL	CIP	CL	FOS	SXT		
SBH193	Patienturine	6265	**>128**	**32**	**64**	0.03	**>128**	**>256**	**>256**	**128**	**4**	**32**	8	**>256**	**>64**	0.25	8	**64**	*tmexCD1*-*toprJ1*, *bla*_SHV-28_, *bla*_TEM-1B_, *bla*_OXA-1_, *bla*_CTX-M-3_, *bla*_DHA-1_, *tet*(A), *aph(3′)-Ia*, *aph(4)-Ia*, *aac(3)-IV*, *aac(3)-IIa*, *aadA1*, *aadA2*, *aadA16*, *strAB*, *armA*, *aac(6′)-Ib-cr*, *oqxAB*, *qnrB1*, *qnrB4*, *catB3*, *cmlA1*, *fosA*, *sul1*, *sul2*, *sul3*, *dfrA14*, *dfrA27*, *mph*(E), *msr*(E), *arr-3*	IncFIB/IncHI1B plasmid
YZ22CK024	Chicken meat	580	**>128**	**128**	0.5	0.03	**>128**	**>256**	**256**	8	**4**	**128**	**>128**	**>256**	**64**	0.25	8	**>64**	*tmexCD1-toprJ1*, *bla*_SHV-11_, *bla*_LAP-2_, *bla*_DHA-1_, *aph(3′)-Ia*, *aph(4)-Ia*, *aac(3)-IV*, *aadA1*, *aadA2*, *aadA8*, *strAB*, *armA*, *oqxAB*, *qnrS1*, *qnrB4*, *cmlA1*, *floR*, *fosA*, *sul1*, *sul2*, *sul3*, *dfrA12*, *mph*(A), *mph*(E), *msr*(E)	IncFIB/IncHI1B plasmid
YZ22CS023	Chicken intestinal contents	1944	**>128**	**>128**	**32**	0.03	**>128**	**>256**	**>256**	**128**	**4**	**128**	**>128**	**>256**	**8**	0.25	**256**	**>64**	*tmexCD1-toprJ1*, *bla*_SHV-11_, *bla*_DHA-1_, *bla*_CTX-M-27_, *tet*(B), *tet*(D), *aac(3)-IId*, *aph(4)-Ia*, *aac(3)-IV*, *aadA1*, *aadA2*, *aadA16*, *strAB*, *armA*, *aac(6′)-Ib-cr*, *oqxAB*, *qnrB4*, *qnrB52*, *cmlA1*, *floR*, *fosA*, *sul1*, *sul3*, *dfrA27*, *msr*(E), *mph*(E), *arr-3*	IncR/ IncFIA/IncFIB/IncHI1B plasmid
YZ22CS070	Chicken intestinal contents	6262	**>128**	**>128**	1	0.03	**>128**	**>256**	**256**	**>128**	**8**	**>128**	**>128**	**>256**	**>64**	**4**	**512**	**64**	*tmexCD1-toprJ1*, *bla*_SHV-11_, *bla*_TEM-1B_, *bla*_DHA-1_, *tet*(D), *aac(3)-IId*, *aph(3′)-Ia*, *aph(4)-Ia*, *aac(3)-IV*, *aadA1*, *aadA2*, *aadA16*, *strAB*, *armA*, *aac(6′)-Ib-cr*, *oqxAB*, *qnrB4*, *qnrB52*, *cmlA1*, *catA2*, *floR*, *fosA*, *sul1*, *sul3*, *dfrA27*, *msr*(E), *mph*(E), *arr-3*	IncFIB(K) plasmid
YZ22CS072	Chicken intestinal contents	6262	**>128**	**>128**	0.25	0.03	**>128**	**>256**	**64**	**>128**	**8**	**>128**	**>128**	**>256**	**>64**	**16**	**>512**	**64**	*tmexCD1-toprJ1*, *bla*_SHV-11_, *bla*_TEM-1B_, *bla*_DHA-1_, *tet*(D), *aac(3)-IId*, *aph(3′)-Ia*, *aph(4)-Ia*, *aac(3)-IV*, *aadA1*, *aadA2*, *aadA16*, *strAB*, *armA*, *aac(6′)-Ib-cr*, *oqxAB*, *qnrB4*, *qnrB52*, *cmlA1*, *catA2*, *floR*, *fosA*, *sul1*, *sul3*, *dfrA27*, *msr*(E), *mph*(E), *arr-3*	IncFIB(K) plasmid
YZ22CS088	Chicken intestinal contents	15	**>128**	**>128**	**64**	0.03	**>128**	**>256**	**128**	**>128**	**8**	**>128**	**>128**	**>256**	**>64**	0.25	16	**64**	*tmexCD1-toprJ1*, *bla*_SHV-28_, *bla*_CTX-M-27_, *bla*_DHA-1_, *tet*(A), *aph(3′)-Ia*, *aph(4)-Ia*, *aac(3)-IV*, *aadA1*, *aadA2*, *aadA16*, *strAB*, *armA*, *aac(6′)-Ib-cr*, *oqxAB*, *qnrB4*, *qnrB52*, *cmlA1*, *floR*, *fosA*, *sul1*, *sul3*, *dfrA27*, *msr*(E), *mph*(E), *arr-3*	IncR/IncFIB(K)/IncHI1B plasmid
YZ22CS089	Chicken intestinal contents	15	**>128**	**>128**	**64**	0.03	**>128**	**>256**	**128**	**>128**	**8**	**>128**	**>128**	**>256**	**>64**	<0.125	16	**64**	*tmexCD1-toprJ1*, *bla*_SHV-28_, *bla*_CTX-M-27_, *bla*_DHA-1_, *tet*(A), *aph(3′)-Ia*, *aph(4)-Ia*, *aac(3)-IV*, *aadA1*, *aadA2*, *aadA16*, *strAB*, *armA*, *aac(6′)-Ib-cr*, *oqxAB*, *qnrB4*, *qnrB52*, *cmlA1*, *floR*, *fosA*, *sul1*, *sul3*, *dfrA27*, *msr*(E), *mph*(E), *arr-3*	IncR/IncFIB(K)/IncHI1B plasmid
YZ22CS094	Chicken intestinal contents	5982	**>128**	**>128**	**32**	0.03	**>128**	**>256**	**128**	**>128**	**4**	**>128**	**>128**	**>256**	**>64**	**4**	16	**64**	*tmexCD1-toprJ1*, *bla*_SHV-11_, *bla*_TEM-1B_, *bla*_CTX-M-3_, *bla*_DHA-1_, *tet*(A), *aph(3′)-Ia*, *aph(4)-Ia*, *aac(3)-IV*, *aadA1*, *aadA2*, *aadA16*, *strAB*, *armA*, *aac(6′)-Ib-cr*, *oqxAB*, *qnrS1*, *qnrB4*, *cmlA1*, *floR*, *fosA*, *sul1*, *sul3*, *dfrA27*, *mph*(A), *msr*(E), *mph*(E), *arr-3*	IncFIB(K)/IncHI1B plasmid
YZ22PK089	Pork	2294	**>128**	**>128**	1	0.03	**>128**	**>256**	**>256**	**128**	**8**	**>128**	**>128**	**>256**	**>64**	**4**	8	**64**	*tmexCD1-toprJ1*, *mcr-1*, *bla*_SHV-1_, *bla*_DHA-1_, *tet*(A), *aph(3′)-Ia*, *aph(4)-Ia*, *aac(3)-IV*, *aac(3)-IId*, *aadA1*, *aadA2*, *aadA16*, *strAB*, *armA*, *aac(6′)-Ib-cr*, *oqxAB*, *qnrB4*, *qnrB52*, *cmlA1*, *floR*, *fosA*, *sul1*, *sul2*, *sul3*, *dfrA27*, *mph*(A), *mph*(E), *msr*(E), *arr-3*	chromosome

a MICs of tigecycline and colistin were determined by broth microdilution method, while others were performed by agar dilution method. The results were interpreted according to CLSI M100, except that streptomycin (≥32 mg/liter), tigecycline (≥1 mg/liter), florfenicol (≥32 mg/liter), and colistin (>2 mg/liter) were interpreted according to EUCAST breakpoints. The MICs values above the breakpoints are show in boldface. Multilocus sequence typing, resistance genes, and plasmid replicons were analyzed using the CGE web-based server. Abbreviations: AMP, ampicillin; CFZ, cefazolin; CTX, cefotaxime; MEM, meropenem; GEN, gentamicin; AMK, amikacin; STR, streptomycin; TET, tetracycline; TIL, tigecycline; CHL, chloramphenicol; FFC, florfenicol; NAL, nalidixic acid; CIP, ciprofloxacin; CL, colistin; FOS, fosfomycin; SXT, sulfamethoxazole-trimethoprim.

**FIG 1:**
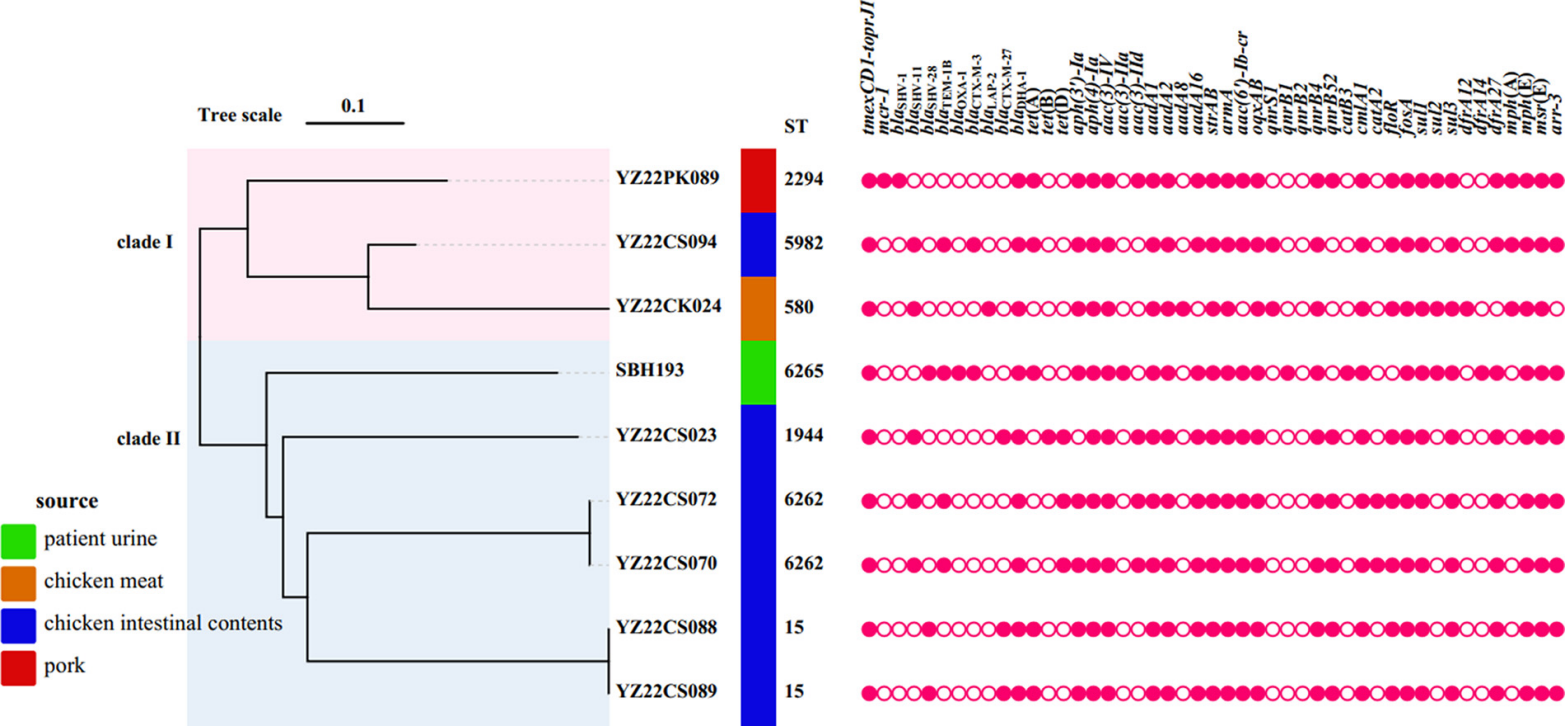
Phylogenetic tree based on core genome SNPs and drug resistance genes of 9 *tmexCD1-toprJ1*-positive Klebsiella pneumoniae strains. Antimicrobial resistance genes are shown in pink solid circles.

Whole-genome sequencing was subsequently performed using Illumina sequencing (NovaSeq) and Oxford Nanopore (MinION system) platforms (GenBank accession number PRJNA893449). The *de novo* hybrid assembly was performed using SPAdes 3.11 (7) and Unicycle version 0.4.9 (8) and corrected using Pilon version 1.23 (9). The genome annotation was performed with Prokka 1.13 (10). *In silico* multilocus sequence typing (MLST) was performed in the Institute Pasteur MLST online database (https://bigsdb.pasteur.fr/klebsiella/). A total of seven STs were identified, namely, ST15 (*n* = 2), ST580, ST1944, ST2294, ST5982, ST6262 (*n* = 2), and ST6265, among which ST6262 and ST6265 were novel STs (Fig. 1, Table 1). Antimicrobial resistance genes and plasmid replicons were analyzed by using the Center for Genomic Epidemiology (CGE) pipeline (http://www.genomicepidemiology.org/). In addition to *tmexCD1-toprJ1*, all nine strains harbored *bla*_DHA-1_, *aph(4)-Ia*, *aac(3)-IV*, *aadA1*, *aadA2*, *strA*/*B*, *armA*, *oqxAB*, *qnrB4*, *cmlA1*, *fosA*, *sul1*, *sul3*, *mph*(E), and *msr*(E). Resistance gene *mcr-1* was identified in YZ22PK089 (Fig. 1, Table 1). The phylogenetic tree was generated using Parsnp (core genome single-nucleotide polymorphism [SNP] tree) (11). Distance matrices describing pairwise allele differences (ignoring missing values) are listed in Table S3. ChiPlot (http://chiplot.online/#) was used to visualize the phylogenetic tree. The nine *tmexCD-toprJ*-positive K. pneumoniae isolates were divided into two clades (Fig. 1). Clade I contained three strains, including one strain from pork, one strain from chicken meat, and one strain from chicken intestinal contents. One strain from a patient and five strains from chicken intestinal contents were classified as clade II; meanwhile, two isolates with the same STs, ST6262 and ST15, were identified in chicken samples, indicating clonal transmission among chicken.

**FIG 2:**
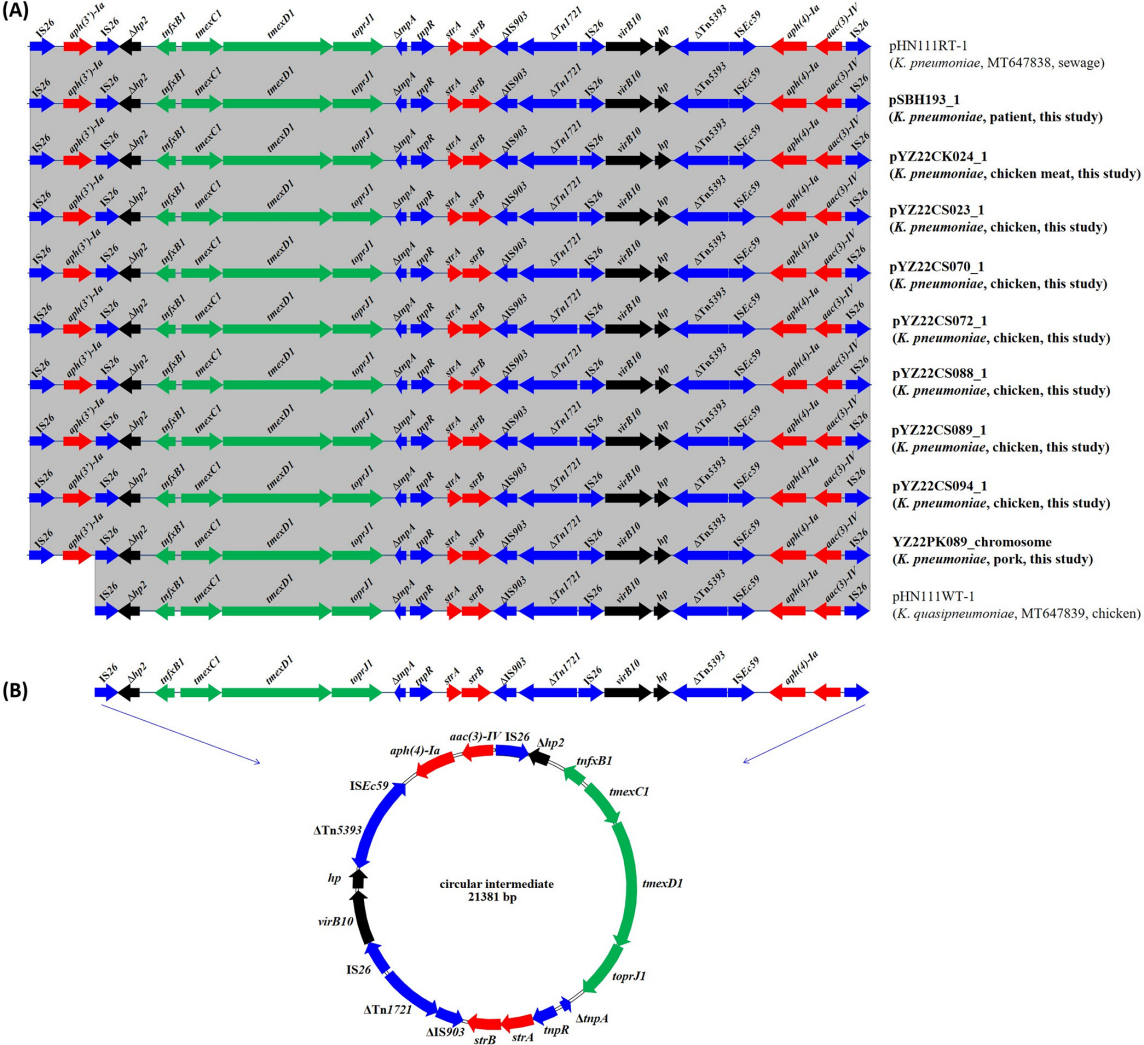
Genetic context structure of *tmexCD1-toprJ1*. (A) Genetic context comparison of *tmexCD1-toprJ1* with closely related sequences. (B) Circular intermediate formation of conserved core structure.

Genomic analysis showed that *tmexCD1-toprJ1* was located on both plasmids (*n* = 8) and the chromosome (*n* = 1) (Table S4). After comparing the sequences of eight plasmids carrying *tmexCD1-toprJ1* (Fig. S1), it was found that only the plasmid pYZ22CK024_1 contained the complete conjugative transfer region, whereas the other plasmids were missing the desired region (pYZ22CS070_1, pYZ22CS072_1, pYZ22CS088_1, pYZ22CS089_1, and pYZ22CS094_1) or had an incomplete conjugation transfer-related gene *TraG* (pSBH193_1 and pYZ22CS023_1). This may explain why only one strain, YZ22CK024, was able to transfer tigecycline resistance to E. coli C600 through conjugation. All the *tmexCD1-toprJ1*-carrying plasmids or chromosome shared a 24,087-bp conserved core structure [IS*26*-*aph(3′)-Ia*-IS*26-*Δ*hp2*-*tnfxB1*-*tmexCD1-toprJ1*-Δ*tnpA*-*tnpR*-*strA*/*B*-ΔIS*903*-ΔTn*1721*-IS*26*-*virB10*-*hp*-ΔTn*5393*-IS*Ec59*-*aph(4)-Ia*-*aac(3)-Iv*-IS*26*], which was identical to that in K. pneumoniae plasmids p18-29-MDR (GenBank accession number MK262712) and pHN111RT-1 (GenBank accession number MT647838) (Fig. 2A). The core structure of *tmexCD1-toprJ1* was also identified in plasmid pHN111WT-1 (*K. quasipneumoniae*; GenBank accession number MT647839), except that the IS*26*-*aph(3′)-Ia* fragment was absent. The 21,381-bp segment [IS*26-*Δ*hp2*-*tnfxB1*-*tmexCD1-toprJ1*-Δ*tnpA*-*tnpR*-*strA*/*B*-ΔIS*903*-ΔTn*1721*-IS*26*-*virB10*-*hp-Δ*Tn*5393*-IS*Ec59*-*aph(4)-Ia*-*aac(3)-Iv*-IS*26*] in pHN111RT-1 and pHN111WT-1 was previously confirmed to form a circular intermediate (Fig. 2B), suggesting that *tmexCD1*-*toprJ1* may be captured by IncHI1B-FIB plasmids through translocatable unit cointegration mediated by IS*26* (12). Our findings further highlight that IS*26* mediates the mobilization of *tmexCD1*-*toprJ1* among different plasmids and chromosomes.

In conclusion, our results showed that *tmexCD1*-*toprJ1* has disseminated among K. pneumoniae strains from poultry, food markets, and patients. IS*26*-mediated horizontal transfer of *tmexCD1*-*toprJ1* could facilitate its rapid and wide dissemination in *Enterobacteriaceae* from various sources. It is critical to strengthen continuous monitoring, and control measures should be implemented to prevent the further dissemination of *tmexCD-toprJ*.

### Ethics statement.

This study was approved by Jiangsu Key Laboratory of Zoonosis and Clinical Medical College, Yangzhou University, Yangzhou, China.

### Data availability.

The genome sequences in this study have been deposited into NCBI GenBank under BioProject accession number PRJNA893449.
