# Supplementary material for: Distribution and Spread of the Mobilized RND Efflux Pump Gene Cluster tmexCD-toprJ in Klebsiella pneumoniae from Different Sources
Source: Microbiol Spectr. 2023 Jun 28;11(4):e05364-22. doi: 10.1128/spectrum.05364-22 (PMC10434155; doi:10.1128/spectrum.05364-22)
Supplement: Supplemental file 1 — Table S1. Download spectrum.05364-22-s0002.docx, DOCX file, 0.02 MB [file spectrum.05364-22-s0002.docx]

**Table S1. *tmexCD-toprJ*-positive *Klebsiella pneumoniae* isolates obtained from different sources in Yangzhou, China**

| **Source** | **Samples** | **No. of**  **colistin resistant isolates (%)** | **No. of**  **tigecycline resistant isolates (%)** | **No. of**  ***tmexCD-toprJ*-positive isotales (%)** |
| --- | --- | --- | --- | --- |
| patients* | 598 | 52 (8.70%) | 245 (40.97%) | 1 (0.17%) |
| healthy human fecal | 309 | 2 (0.65%) | 2 (0.65%) | 0 |
| pet fecal | 206 | 3 (1.46%) | 5 (2.43%) | 0 |
| pork | 117 | 2 (1.71%) | 19 (16.24%) | 1 (0.85%) |
| pig fecal | 35 | 0 | 0 | 0 |
| chicken meat | 118 | 1 (0.85%) | 4 (3.39%) | 1 (0.85%) |
| chicken intestinal contents | 158 | 4 (2.53%) | 28 (17.72%) | 6 (3.80%) |
| total | 1541 | 64 (4.15%) | 303 (19.66%) | 9 (0.58%) |

*598 clinical *K. pneumoniae* isolates were collected from 2 tertiary hospitals in Yangzhou.
