# Supplementary material for: Distribution and Spread of the Mobilized RND Efflux Pump Gene Cluster tmexCD-toprJ in Klebsiella pneumoniae from Different Sources
Source: Microbiol Spectr. 2023 Jun 28;11(4):e05364-22. doi: 10.1128/spectrum.05364-22 (PMC10434155; doi:10.1128/spectrum.05364-22)
Supplement: Supplemental file 2 — Table S2. Download spectrum.05364-22-s0003.docx, DOCX file, 0.01 MB [file spectrum.05364-22-s0003.docx]

**Table S2. MICs of Tigecycline against *tmexCD1-toprJ1*-carrying transconjugant and recipient**

| **Strains** | **Tigecycline MIC (mg/L)** |
| --- | --- |
| *E. coli* C600 | 0.06 |
| Transconjugant C600-YZ22CK024 | 4 |
