# Supplementary material for: Distribution and Spread of the Mobilized RND Efflux Pump Gene Cluster tmexCD-toprJ in Klebsiella pneumoniae from Different Sources
Source: Microbiol Spectr. 2023 Jun 28;11(4):e05364-22. doi: 10.1128/spectrum.05364-22 (PMC10434155; doi:10.1128/spectrum.05364-22)
Supplement: Supplemental file 3 — Table S3. Download spectrum.05364-22-s0004.docx, DOCX file, 0.02 MB [file spectrum.05364-22-s0004.docx]

**Table S3. cgMLST distance matrices for pairwise comparisons of 9 *tmexCD-toprJ*-positive *Klebsiella pneumoniae* isolates in this study**

|  | **YZ22CS094** | **YZ22CS072** | **YZ22CS023** | **YZ22CS070** | **YZ22PK089** | **YZ22CS089** | **SBH193** | **YZ22CK024** | **YZ22CS088** |
| --- | --- | --- | --- | --- | --- | --- | --- | --- | --- |
| **YZ22CS094** | 0 | 27369 | 28387 | 27362 | 18609 | 28601 | 27301 | 16464 | 28602 |
| **YZ22CS072** | 27369 | 0 | 27669 | 7 | 28912 | 27833 | 27890 | 28270 | 27832 |
| **YZ22CS023** | 28387 | 27669 | 0 | 27664 | 28474 | 28510 | 27259 | 27875 | 28509 |
| **YZ22CS070** | 27362 | 7 | 27664 | 0 | 28911 | 27828 | 27883 | 28265 | 27827 |
| **YZ22PK089** | 18609 | 28912 | 28474 | 28911 | 0 | 29121 | 27931 | 29213 | 29122 |
| **YZ22CS089** | 28601 | 27833 | 28510 | 27828 | 29121 | 0 | 28233 | 29632 | 7 |
| **SBH193** | 27301 | 27890 | 27259 | 27883 | 27931 | 28233 | 0 | 27436 | 28232 |
| **YZ22CK024** | 16464 | 28270 | 27875 | 28265 | 29213 | 29632 | 27436 | 0 | 29633 |
| **YZ22CS088** | 28602 | 27832 | 28509 | 27827 | 29122 | 7 | 28232 | 29633 | 0 |
