# Supplementary material for: Distribution and Spread of the Mobilized RND Efflux Pump Gene Cluster tmexCD-toprJ in Klebsiella pneumoniae from Different Sources
Source: Microbiol Spectr. 2023 Jun 28;11(4):e05364-22. doi: 10.1128/spectrum.05364-22 (PMC10434155; doi:10.1128/spectrum.05364-22)
Supplement: Supplemental file 4 — Table S4. Download spectrum.05364-22-s0005.docx, DOCX file, 0.02 MB [file spectrum.05364-22-s0005.docx]

**Table S4. Whole genomes of *tmexCD1-toprJ1*-positive *Klebsiella penumoniae* isolates in this study**

|  | **Size**  **(bp)** | **Resistance genes** | **Plasmid replicon** |
| --- | --- | --- | --- |
| **SBH193** |  |  |  |
| chromosome | 5,465,190 | *bla*_SHV-28_, *oqxAB*, *fosA* | - |
| pSBH193_1 | 344,396 | *tmexCD1*-*toprJ1*, *bla*_DHA-1_, *aph(3')-Ia*, *aph(4)-Ia*, *aac(3)-IV*, *aadA1*, *aadA2*, *aadA16*, *strA*/*B*, *armA*, *qnrB4*, *cmlA1*, *sul1*, *sul3*, *dfrA27*, *mph*(E), *msr*(E), *arr-3* | IncFIB/IncHI1B |
| pSBH193_2 | 238,814 | *bla*_TEM-1B_, *bla*_OXA-1_, *bla*_CTX-M-3_, *tet*(A), *aac(3)-IIa*, *strA*/*B*, *aac(6')-Ib-cr*, *qnrB1*, *catB3*, *sul2*, *dfrA14* | IncFII(K)/IncFIB(K) |
| **YZ22CK024** |  |  |  |
| chromosome | 5,304,021 | *bla*_SHV-11_, *aph(4)-Ia*, *aac(3)-IV*, *aadA8*, *oqxAB*, *floR*, *fosA*, *sul2*, *sul3*, *dfrA12* | - |
| pYZ22CK024_1 | 273,252 | *tmexCD1-toprJ1*, *bla*_DHA-1_, *aph(3')-Ia*, *aph(4)-Ia*, *aac(3)-IV*, *aadA1*, *aadA2*, *strA*/*B*, *armA*, *qnrB4*, *cmlA1*, *sul1*, *sul3*, *mph*(E), *msr*(E) | IncFIB/IncHI1B |
| pYZ22CK024_2 | 216,138 | *bla*_LAP-2_, *aph(3')-Ia*, *aadA2*, *qnrS1*, *sul1*, *sul2*, *dfrA12*, *mph*(A) | IncFIB(K) |
| **YZ22CS023** |  |  |  |
| chromosome | 5,270,699 | *bla*_SHV-11_, *oqxAB*, *fosA* | - |
| pYZ22CS023_1 | 368,144 | *tmexCD1-toprJ1*, *bla*_DHA-1_, *bla*_CTX-M-27_, *tet*(B), *tet*(D), *aac(3)-IId*, *aph(4)-Ia*, *aac(3)-IV*, *aadA1*, *aadA2*, *aadA16*, *strA*/*B*, *armA*, *aac(6')-Ib-cr*, *qnrB4*, *qnrB52*, *cmlA1*, *floR*, *sul1*, *sul3*, *dfrA27*, *msr*(E), *mph*(E), *arr-3* | IncR/ IncFIA/IncFIB/IncHI1B |
| **YZ22CS070** |  |  |  |
| chromosome | 5,200,817 | *bla*_SHV-11_, *oqxAB*, *fosA* | - |
| pYZ22CS070_1 | 224,600 | *tmexCD1-toprJ1*, *bla*_TEM-1B_, *bla*_DHA-1_, *aac(3)-IId*, *aph(3')-Ia*, *aph(4)-Ia*, *aac(3)-IV*, *aadA1*, *aadA2*, *strA*/*B*, *armA*, *qnrB4*, *cmlA1*, *sul1*, *sul3*, *msr*(E), *mph*(E) | IncFIB(K) |
| pYZ22CS070_2 | 119,654 | *bla*_TEM-1B_, *tet*(D), *aadA16*, *aac(6')-Ib-cr*, *qnrB52*, *catA2*, *floR*, *sul1*, *dfrA27*, *arr-3* | IncR/IncFIA |
| **YZ22CS072** |  |  |  |
| chromosome | 5,200,907 | *bla*_SHV-11_, *oqxAB*, *fosA* | - |
| pYZ22CS072_1 | 224,600 | *tmexCD1-toprJ1*, *bla*_TEM-1B_, *bla*_DHA-1_, *aac(3)-IId*, *aph(3')-Ia*, *aph(4)-Ia*, *aac(3)-IV*, *aadA1*, *aadA2*, *strA*/*B*, *armA*, *qnrB4*, *cmlA1*, *sul1*, *sul3*, *msr*(E), *mph*(E) | IncFIB(K) |
| pYZ22CS072_2 | 119,650 | *bla*_TEM-1B_, *tet*(D), *aadA16*, *aac(6')-Ib-cr*, *qnrB52*, *catA2*, *floR*, *sul1*, *dfrA27*, *arr-3* | IncR/IncFIA |
| **YZ22CS088** |  |  |  |
| chromosome | 5,457,787 | *bla*_SHV-28_, *oqxAB*, *fosA* | - |
| pYZ22CS088_1 | 239,545 | *tmexCD1-toprJ1*, *bla*_DHA-1_, *aph(3')-Ia*, *aph(4)-Ia*, *aac(3)-IV*, *aadA1*, *aadA2*, *strA*/*B*, *armA*, *qnrB4*, *cmlA1*, *sul1*, *sul3*, *msr*(E), *mph*(E) | IncR/IncFIB(K)/IncHI1B |
| pYZ22CS088_2 | 60,163 | *bla*_CTX-M-27_, *tet*(A), *aadA16*, *aac(6')-Ib-cr*, *qnrB52*, *sul1*, *dfrA27*, *arr-3* | IncR/IncFIA |
| pYZ22CS088_3 | 29,087 | *floR* | IncX1 |
| pYZ22CS088_4 | 5,831 | - | - |
| pYZ22CS088_5 | 3,478 | - | - |
| pYZ22CS088_6 | 2,058 | - | ColpVC |
| **YZ22CS089** |  |  |  |
| chromosome | 5,455,592 | *bla*_SHV-28_, *oqxAB*, *fosA* | - |
| pYZ22CS089_1 | 239,545 | *tmexCD1-toprJ1*, *bla*_DHA-1_, *aph(3')-Ia*, *aph(4)-Ia*, *aac(3)-IV*, *aadA1*, *aadA2*, *strA*/*B*, *armA*, *qnrB4*, *cmlA1*, *sul1*, *sul3*, *msr*(E), *mph*(E) | IncR/IncFIB(K)/IncHI1B |
| pYZ22CS089_2 | 60,381 | *bla*_CTX-M-27_, *tet*(A), *aadA16*, *aac(6')-Ib-cr*, *qnrB52*, *sul1*, *dfrA27*, *arr-3* | IncR/IncFIA |
| pYZ22CS089_3 | 30,049 | *floR* | IncX1 |
| pYZ22CS089_4 | 5,831 | - | - |
| pYZ22CS089_5 | 3,478 | - | - |
| pYZ22CS089_6 | 2,058 | - | ColpVC |
| **YZ22CS094** |  |  |  |
| chromosome | 5,345,654 | *bla*_SHV-11_, *oqxAB*, *fosA* | - |
| pYZ22CS094_1 | 262,096 | *tmexCD1-toprJ1*, *bla*_DHA-1_, *aph(3')-Ia*, *aph(4)-Ia*, *aac(3)-IV*, *aadA1*, *aadA2*, *strA*/*B*, *armA*, *qnrB4*, *cmlA1*, *sul1*, *sul3*, *msr*(E), *mph*(E) | IncFIB(K)/IncHI1B |
| pYZ22CS094_2 | 131,266 | *bla*_TEM-1B_, *bla*_CTX-M-3_, *tet*(A), *aac(6')-Ib-cr*, *aadA16*, *qnrS1*, *floR*, *sul1*, *dfrA27*, *mph*(A), *arr-3* | IncFII(K) |
| **YZ22PK089** |  |  |  |
| chromosome | 5,295,183 | *tmexCD1-toprJ1*, *bla*_SHV-1_, *bla*_DHA-1_, *aph(3')-Ia*, *aph(4)-Ia*, *aac(3)-IV*, *aadA1*, *aadA2*, *strA*/*B*, *armA*, *qnrB4*, *oqxAB*, *cmlA1*, *fosA*, *sul1*, *sul3*, *mph*(E), *msr*(E) | - |
| pYZ22PK089_1 | 171,542 | *tet*(A), *aph(3')-Ia*, *aac(3)-IId*, *aadA16*, *strA*/*B*, *aac(6')-Ib-cr*, *qnrB52*, *floR*, *mph*(A), *sul1*, *sul2*, *dfrA27*, *arr-3* | IncQ1/IncFII(K)/IncFIB(K) |
| pYZ22PK089_2 | 33,309 | *mcr-1* | IncX4 |
| pYZ22PK089_3 | 5,459 | - | Col(pHAD28) |
| pYZ22PK089_4 | 3,809 | - | Col440I |
| pYZ22PK089_5 | 3,086 | - | - |
