# Supplementary material for: Distribution and Spread of the Mobilized RND Efflux Pump Gene Cluster tmexCD-toprJ in Klebsiella pneumoniae from Different Sources
Source: Microbiol Spectr. 2023 Jun 28;11(4):e05364-22. doi: 10.1128/spectrum.05364-22 (PMC10434155; doi:10.1128/spectrum.05364-22)
Supplement: Supplemental file 5 — Fig. S1. Download spectrum.05364-22-s0001.docx, DOCX file, 0.4 MB [file spectrum.05364-22-s0001.docx]

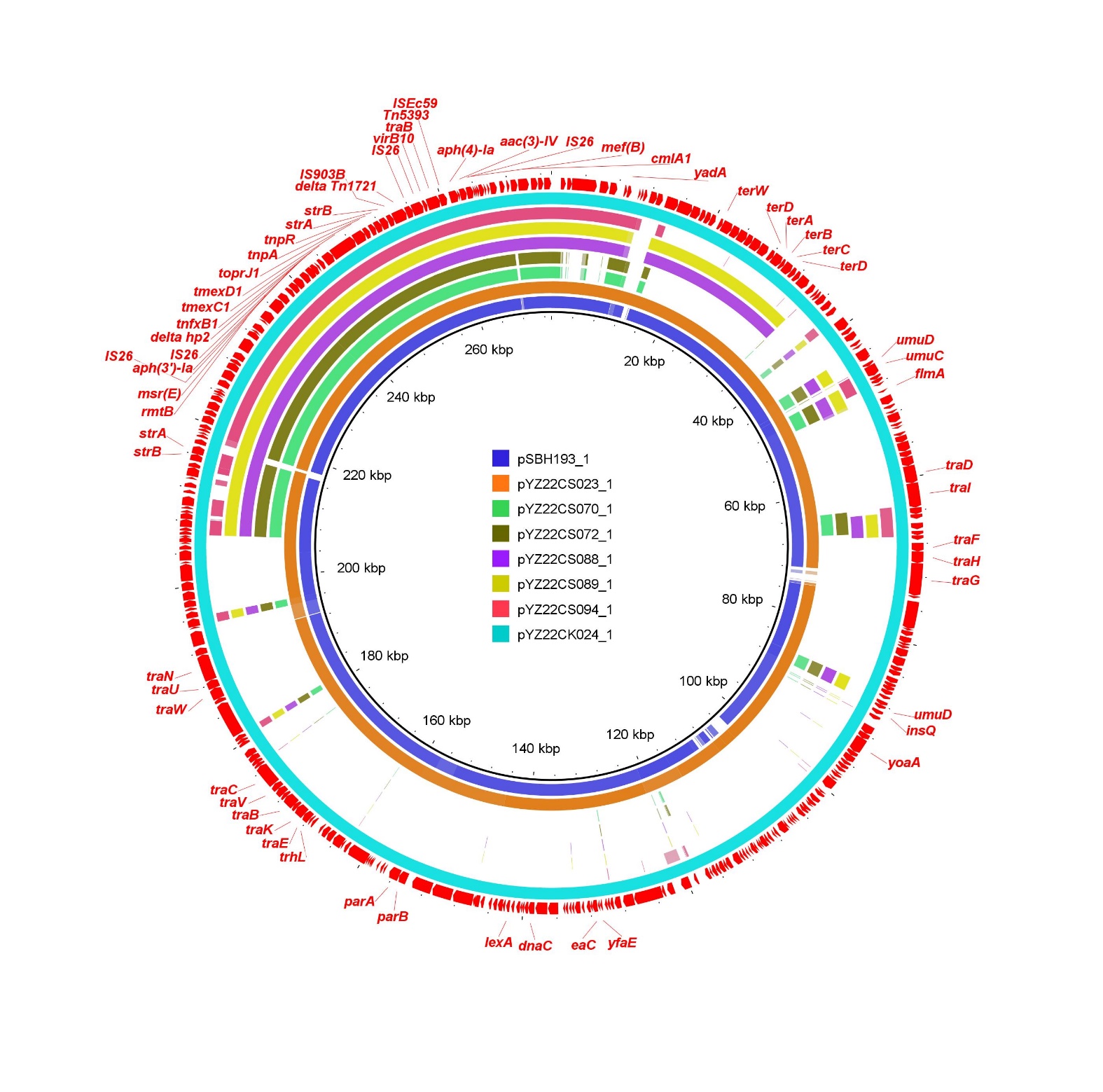


Figure S1. Sequence comparison of the *tmexCD1-toprJ1*-carrying plasmids in this study. Circles from the inside to outside indicate plasmids of pSBH193_1 (CP110143), pYZ22CS023_1 (CP110149), pYZ22CS070_1 (CP110266), pYZ22CS072_1 (CP110151), pYZ22CS088_1 (CP110154), pYZ22CS089_1 (CP110161), pYZ22CS094_1 (CP110168), and pYZ22CK024_1 (CP110146). Arrows indicate the positions and the direction of gene transcription.
